# Clinical Outcome of Endoscopic Transpapillary Drainage for Biliary Obstruction Due to Non-Hepato-Pancreato-Biliary Cancer: A Two-Center Retrospective Cohort Study

**DOI:** 10.3390/clinpract16020024

**Published:** 2026-01-23

**Authors:** Kensuke Kitsugi, Kazuhito Kawata, Yoshisuke Hosoda, Yashiro Yoshizawa, Masaharu Kimata, Yosuke Kobayashi, Shuhei Unno, Yosuke Yamada, Hidenao Noritake, Takeshi Chida, Go Murohisa

**Affiliations:** 1 Department of Gastroenterology, Seirei Hamamatsu General Hospital, Shizuoka 430-8558, Japan; yhosoda@sis.seirei.or.jp (Y.H.); yashiro1224@sis.seirei.or.jp (Y.Y.); masa-kimata@sis.seirei.or.jp (M.K.); koba0706@msn.com (Y.K.); shuhei.sora@gmail.com (S.U.); yosuke.h.smile@gmail.com (Y.Y.); murohisa@sis.seirei.or.jp (G.M.); 2 Department of Internal Medicine II, Hamamatsu University School of Medicine, Shizuoka 431-3192, Japan; kawata@hama-med.ac.jp (K.K.); noritake@hama-med.ac.jp (H.N.); tchida@hama-med.ac.jp (T.C.)

**Keywords:** C-reactive protein, endoscopic retrograde cholangiopancreatography, metastasis, obstructive jaundice, serum albumin

## Abstract

**Objective**: Although non-hepato-pancreato-biliary (non-HPB) cancer, such as gastric and colorectal cancer, may cause biliary obstruction, the efficacy of endoscopic transpapillary drainage remains unclear. We investigated the clinical outcomes of endoscopic transpapillary drainage for biliary obstruction due to non-HPB cancer. **Methods**: This was a two-center retrospective observation study. We evaluated the technical success, clinical success, recurrent biliary obstruction (RBO), time to RBO (TRBO), adverse events (AEs), and overall survival (OS). OS was determined using the Kaplan–Meier method, and the significance was tested using the log-rank test. Cox regression hazard models were performed to identify the independent association of clinical parameters with OS. **Results**: This study included 43 cases. The technical success was achieved in all cases (100%), and the clinical success was achieved in 35 cases (81%). The occurrence rate of RBO and non-RBO AEs were 33% and 12%, respectively. The median TRBO was 176 days. Systemic chemotherapy was introduced in 17 cases (40%) after biliary drainage, and cases with the introduction of systemic chemotherapy had a significantly longer OS. C-reactive protein <3.4 mg/dL and biliary obstruction due to lymph node metastasis were independently associated with the introduction of systemic chemotherapy. In survival analysis, serum albumin >3.1 g/dL and the introduction of systemic chemotherapy were significant and independent predictive factors for the prolongation of OS. **Conclusions**: The endoscopic transpapillary drainage for biliary obstruction due to non-HPB cancer can provide favorable outcomes with appropriate patient selection.

## 1. Introduction

Hepatobiliary and pancreatic malignant tumors often cause biliary obstruction. Biliary obstruction causes obstructive jaundice and deteriorates the prognosis. Therefore, appropriate biliary drainage is required [1]. Transpapillary biliary drainage with endoscopic retrograde cholangiopancreatography (ERCP) is the gold standard for biliary decompression and provides symptomatic relief [2].

Non-hepato-pancreato-biliary (non-HPB) cancer, such as gastric, colorectal, and breast cancer, also causes biliary obstruction [3,4,5]. Unlike HPB cancer, biliary obstruction due to non-HPB cancer is caused by lymph node metastasis, liver metastasis, and peritoneal dissemination. Therefore, most patients are found to be incurable and have a poorer prognosis than HPB cancer [6]. To improve the prognosis, systemic chemotherapy should be initiated after the alleviation of jaundice by biliary drainage. Previous studies on biliary drainage for biliary obstruction due to non-HPB cancer are limited, as they are mainly case reports. Yuan et al. investigated 60 cases with obstructive jaundice secondary to metastatic cancer and reported that biliary drainage was a safe and effective treatment method [6]. Similarly, Van Laethem et al. reported that endoscopic biliary drainage for biliary obstruction due to metastatic cancer resolved jaundice in 86% and improved overall survival [7]. On the other hand, Nichols et al. investigated 62 cases with obstructive jaundice due to colorectal cancer, and reported that biliary decompression has little effect and prognosis is extremely poor [8]. Kastelijn et al. also reported that successful biliary drainage for biliary obstruction due to metastatic colorectal cancer is challenging to achieve and frequently associated with adverse events [9]. Okamoto reviewed malignant biliary obstruction due to metastatic non-HPB cancer and reported that most studies focused on percutaneous drainage [10]. Thus, biliary obstruction due to non-HPB cancer is extremely complex, with differences in the cause of obstruction and primary cancer type, and there is no established opinion regarding the efficacy of biliary drainage.

This study investigated the clinical outcomes of transpapillary drainage using ERCP for biliary obstruction due to non-HPB cancer.

## 2. Materials and Methods

### 2.1. Patients

**Figure 1:**
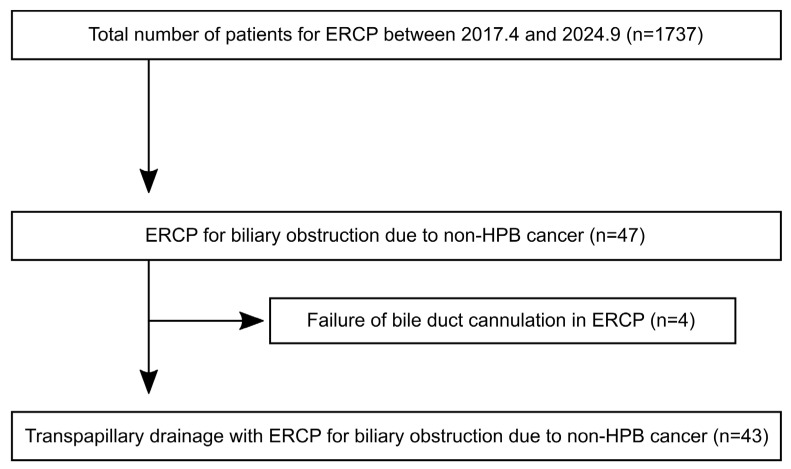
Flow diagram of the study. ERCP, endoscopic retrograde cholangiopancreatography; HPB, hepato-pancreato-biliary.

This was a retrospective observation study. The study population consisted of patients who underwent transpapillary biliary drainage using ERCP for biliary obstruction due to non-HPB cancer at Seirei Hamamatsu General Hospital and Hamamatsu University Hospital between April 2017 and September 2024. Specifically, biliary obstruction due to non-HPB cancer was defined as biliary obstruction due to peritoneal dissemination or metastatic lesions associated with malignant tumors other than hepatocellular carcinoma, biliary tract cancer, and pancreatic cancer. Biliary obstruction was diagnosed by computed tomography or magnetic resonance imaging. The inclusion criteria were as follows: (1) aged > 18 years, (2) cases that have difficulty in introduction of systemic chemotherapy or have symptoms due to abnormalities in hepatobiliary enzyme levels in laboratory data. Cases of switching to other drainage procedures due to failed ERCP were excluded. The patient selection process is summarized in Figure 1. This study was approved by the Ethics Committee of Hamamatsu University Hospital (Approval Number: 24-236), and performed in accordance with the Declaration of Helsinki. The need for informed consent was waived by the Ethics Committee of Hamamatsu University Hospital due to the retrospective study. Each patient was offered the opportunity to decline participation in the study through an opt-out option.

### 2.2. Procedures

ERCP was performed using a side-viewing endoscope (JF-260V and TJF-Q290V; Olympus Medical Systems, Tokyo, Japan). A SIF-H290S endoscope (Olympus Medical Systems, Tokyo, Japan) was used in two cases because of surgically altered anatomy. All procedures were performed with standard cannulation techniques by experts or by trainees under their direct guidance. All cases were sedated with intravenous midazolam. Cases were implanted with either 7-Fr plastic stents (PS) or self-expandable metal stents (SEMS) at the discretion of the endoscopists. In the case of SEMS, an 8 mm uncovered SEMS (UCSEMS) was placed above the papilla in cases of hilar biliary obstruction, and a 10 mm fully covered SEMS (FCSEMS) was placed across the papilla in distal biliary obstruction. In cases of hilar biliary obstruction, bilateral endoscopic biliary drainage was attempted depending on the necessity. Endoscopic sphincterotomy (EST) with medium incision size was performed at the sole discretion of the endoscopist in cases where stents were placed across the papilla.

### 2.3. Outcome Measurements and Definitions

The primary endpoint of this study was to evaluate the efficacy and safety of transpapillary drainage using ERCP for biliary obstruction due to non-HPB cancer. The technical success, clinical success, recurrent biliary obstruction (RBO), time to RBO (TRBO), and non-RBO adverse events (AEs) were investigated to evaluate the efficacy and safety of biliary drainage. The definitions of these evaluation items were based on “the TOKYO criteria 2024” [11]. Technical success was defined as successful stent placement in the intended location of the bile duct. Clinical success was defined as a ≥50% reduction or normalization of total bilirubin for cases with jaundice, a ≥50% reduction or normalization of hepatobiliary enzyme levels for cases without jaundice. The safety of the procedures was assessed by RBO and non-RBO AEs. RBO was defined as stent occlusion or migration requiring biliary drainage or stent removal after the achievement of technical and clinical successes. TRBO was defined as the time between the initial stenting and the occurrence of RBO. Non-RBO AEs included pancreatitis, non-occlusion cholangitis, cholecystitis, liver abscess, bleeding, perforation of the gastrointestinal tract, bile leak, peritonitis. The secondary endpoint was to investigate the factors associated with overall survival (OS) and the introduction of systemic chemotherapy. OS was defined as the time from the biliary drainage until death from any cause.

### 2.4. Statistical Analyses

Data on patient characteristics are presented as numbers for categorical data and medians and full ranges for continuous variables. Differences between paired groups were analyzed using the Wilcoxon signed-rank test. The Mann–Whitney U test was used to compare independent samples. Categorical variables were compared between independent samples using Fisher’s exact test. Univariate and multivariate analyses were performed using a logistic regression model for predicting the introduction of systemic chemotherapy. OS was determined using the Kaplan–Meier method, and the significance was tested using the log-rank test. Cox regression hazard models were performed to identify the independent association of clinical parameters with OS. The cut-off values for laboratory data in survival analysis, univariate analysis, and multivariate analysis were the median value of all cases. One-way analysis of variance followed by Bonferroni’s post hoc test to compare the means of three or more samples. All analyses were performed using EZR, a modified version of the R commander designed to add statistical functions frequently used in biostatistics [12]. A *p*-value of <0.05 was considered statistically significant.

## 3. Results

### 3.1. Patient Characteristics

Of the 1737 ERCP procedures performed during this period, biliary obstruction due to non-HPB cancer was observed in 47 cases (2.7%). Since transpapillary drainage had to be abandoned in four cases due to difficult biliary cannulation, 43 cases were eventually evaluated in this study. The baseline patient characteristics are summarized in Table 1. Seventy percent of the cases had a poor general condition with an Eastern Cooperative Oncology Group Performance Status (ECOG-PS) of two or higher. The most common primary tumor was colorectal cancer (18 cases, 42%), and the most common site of metastasis that caused biliary obstruction was peritoneal dissemination (14 cases, 33%). Duodenal invasion was observed in 16 cases (37%). Regarding the obstruction site, distal biliary obstruction was 27 cases (63%) and hilar biliary obstruction was 16 cases (37%). Twenty-two cases (51%) developed biliary obstruction during systemic chemotherapy. There were no cases of acute cholangitis. The median total bilirubin level at baseline was 7.0 mg/dL. The bilirubin level was normal (<2.0 mg/dL) in four cases, but all had elevated hepatobiliary enzyme levels, making it difficult to introduce systemic chemotherapy. Biliary drainage was attempted more than once in 17 cases due to poor drainage (six cases, 14%) or RBO (11 cases, 26%). For biliary drainage, PS was selected in 27 cases (63%) and SEMS in 16 cases (37%). Bilateral endoscopic biliary drainage was attempted in seven cases of hilar biliary obstruction; however, the obstruction was severe in two cases, and unilateral drainage was performed.

### 3.2. Clinical Outcomes

**Figure 2:**
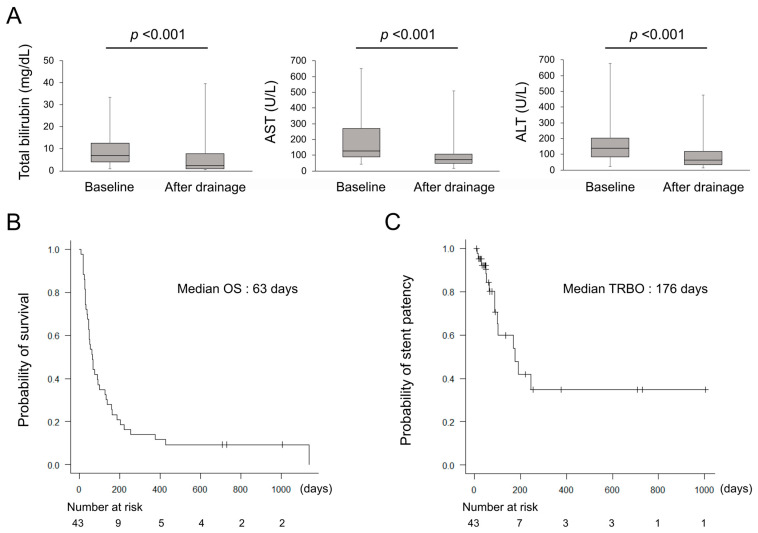
(**A**) The changes in serum total bilirubin, AST, and ALT levels by biliary drainage. (**B**) Kaplan–Meier curves of OS. (**C**) Kaplan–Meier curves of TRBO. ALT, alanine aminotransferase; AST, aspartate aminotransferase; OS, overall survival; TRBO, time to recurrent biliary obstruction.

**Figure 3:**
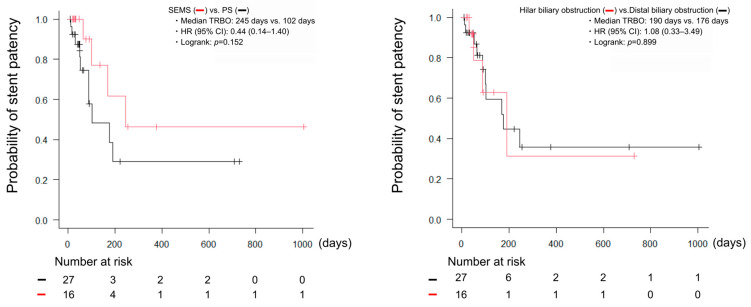
Kaplan–Meier curves of TRBO stratified by the stent type or the obstruction site. CI, confidence interval; HR, hazard ratio; TRBO; PS, plastic stents; SEMS, self-expandable metal stents; TRBO, time to recurrent biliary obstruction.

The technical and clinical success, RBO, non-RBO AEs, and OS are shown in Table 2. The technical success was achieved in all cases (100%). The serum bilirubin, aspartate aminotransferase (AST), and alanine aminotransferase (ALT) levels significantly improved after biliary drainage (Figure 2A). The clinical success was achieved in 35 cases (81%). No significant differences were observed in the patient characteristics between the clinical success group and the non-clinical success group (Table 3). Regarding the safety of the procedures, RBO occurred in 14 cases (33%), and the causes of RBO were tumor ingrowth in five cases (12%), tumor overgrowth in five cases (12%), and sludge in four cases (9%). Non-RBO AEs occurred in five cases (12%), all of which were mild pancreatitis and resolved with conservative treatment. Figure 2B shows the Kaplan–Meier curves for OS and TRBO. The median OS was 63 days (8–1140 days), and 26 cases (60%) died without RBO. The median TRBO was 176 days (11–245 days), and the non-obstruction rates at three and six months were 71% and 48%, respectively. There were no significant differences in TRBO based on stent type or obstruction site (Figure 3).

### 3.3. Analysis of the Factors Contributing to the Introduction of Systemic Chemotherapy After Biliary Drainage and the Prolongation of OS

**Figure 4:**
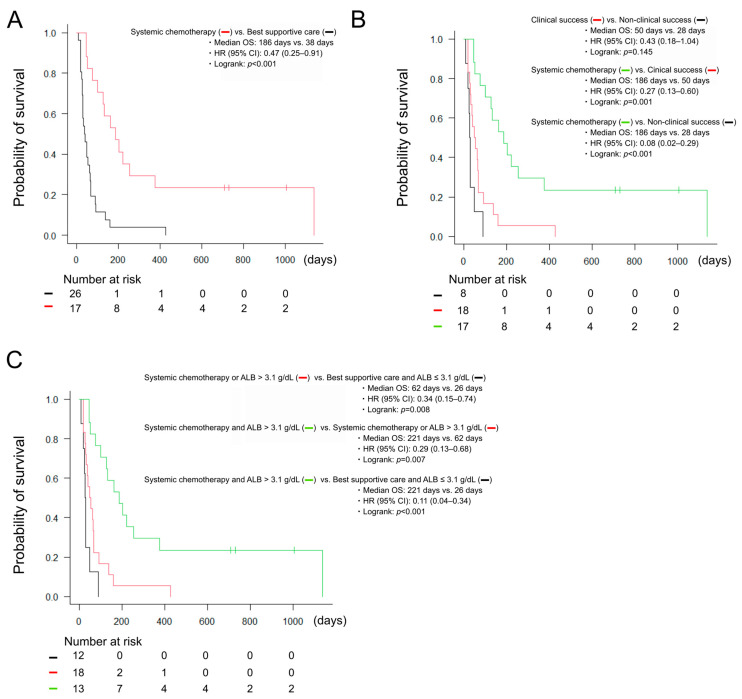
(**A**) Kaplan–Meier curve of OS stratified by the treatment status after biliary drainage. (**B**) Kaplan–Meier curve of OS in the introduction of systemic chemotherapy group, the clinical success group, and the non-clinical success group. (**C**) Kaplan–Meier curve of OS stratified by using serum albumin levels and the treatment status after biliary drainage. ALB, albumin; CI, confidence interval; HR, hazard ratio; OS, overall survival.

The introduction of systemic chemotherapy after biliary drainage was achieved in 17 cases (40%), accounting for 49% of cases with clinical success. Moreover, survival analysis using the Kaplan–Meier method revealed that the cases with the introduction of systemic chemotherapy showed a significant prolongation of OS (Figure 4A). Furthermore, the cases with the introduction of systemic chemotherapy showed a significant prolongation of OS even when compared with the cases with clinical success (Figure 4B). Univariate and multivariate analyses for predictive factors of the introduction of systemic chemotherapy are shown in Table 4. In univariate analysis, biliary obstruction due to lymph node metastasis (OR = 4.89, *p* = 0.030), distal biliary obstruction (OR = 4.67, *p* = 0.039), and serum C-reactive protein (CRP) levels <3.4 mg/dL (OR = 4.27, *p* = 0.032) were significantly associated with the introduction of systemic chemotherapy. In multivariate analysis, biliary obstruction due to lymph node metastasis (OR = 8.71, *p* = 0.023) and serum CRP levels <3.4 mg/dL (OR = 8.62, *p* = 0.016) were independently associated with the introduction of systemic chemotherapy. Comparing the high CRP (≥3.4 mg/dL) and the low CRP (<3.4 mg/dL) groups, there was no significant difference in the clinical success rate (86% vs. 81%, *p* = 1.000) and the normalization of serum bilirubin levels (67% vs. 71%, *p* = 1.000), whereas serum albumin levels were significantly lower in the high CRP group (Median 2.7 g/dL vs. 3.3 g/dL, *p* < 0.001), suggesting poorer general condition in cases with high CRP levels. In case with biliary obstruction due to lymph node metastasis, while there were no differences in serum albumin levels (Median 3.0 g/dL vs. 3.1 g/dL, *p* = 0.924), the clinical success rate (100% vs. 74%, *p* = 0.082) and the normalization of serum bilirubin levels (92% vs. 58%, *p* = 0.067) tended to be higher. In the survival analysis, univariate analysis revealed that serum alkaline phosphatase (ALP) levels <736 U/L (HR = 0.52, *p* = 0.046), serum albumin levels >3.1 g/dL (HR = 0.40, *p* = 0.007), serum CRP levels <3.4 mg/dL (HR = 0.46, *p* = 0.018), and the introduction of systemic chemotherapy (HR = 0.24, *p* < 0.001) were significantly associated with the prolongation of OS, and multivariate analysis revealed that serum albumin levels >3.1 g/dL (HR = 0.44, *p* = 0.026) and the introduction of systemic chemotherapy (HR = 0.23, *p* < 0.001) were independently associated with the prolongation of OS (Table 5). It was possible to stratify the prognosis of cases with non-HPB cancer who underwent transpapillary biliary drainage by using serum albumin levels and the treatment status after biliary drainage (Figure 4C).

## 4. Discussion

In the present study, we demonstrated that transpapillary drainage with ERCP is effective and safe for biliary obstruction due to non-HPB cancer. Moreover, the cases with high serum albumin levels and the introduction of systemic chemotherapy showed a significant prolongation of OS. Since the purpose of biliary drainage for malignant biliary obstruction is to improve prognosis, this study is meaningful in that it investigated the factors contributing to the prolongation of OS. Advances in molecular targeted therapy and immunotherapy have improved the prognosis of various cancer types. Therefore, it is expected that the opportunities to perform biliary drainage for biliary obstruction due to non-HPB cancer will increase. However, the clinical success rate and OS in our study were not satisfactory. In the previous reports, the clinical success rate of biliary drainage for biliary obstruction due to HPB cancer has been reported to be 95–100%, and the median OS after biliary drainage was 150–239 days [13,14,15,16,17]. Therefore, it is suggested that the treatment outcomes of biliary drainage for biliary obstruction due to non-HPB cancer may be poorer than for biliary obstruction due to HPB cancer. Considering that the cases with biliary obstruction due to non-HPB cancer have a poor prognosis and the complications of ERCP are sometimes fatal, it is important to identify the cases in which biliary drainage is effective.

The present study revealed a significant prolongation of OS in cases with high serum albumin levels (>3.1 g/dL) at baseline and the introduction of systemic chemotherapy after drainage. Serum albumin plays an important role through the anti-inflammatory and antioxidant effects in various types of cancer, including non-HPB cancer [18]. Moreover, hypoalbuminemia in cases with unresectable malignant biliary obstruction has been associated with poor OS [19] and serum albumin has been reported as a prognostic factor for cases undergoing biliary drainage for malignant biliary obstruction [20]. In a previous study of biliary drainage for non-HPB cancer, high serum albumin levels (>3.5 g/dL) were associated with prolonged OS [6], as in our study. These findings suggest the importance of nutritional management in biliary drainage for malignant biliary obstruction. Likewise, the introduction of systemic chemotherapy is crucial for improving the prognosis of cases with unresectable cancer. Several studies have reported that the introduction of systemic chemotherapy after biliary drainage is the key to long-term survival [6,8,10]. In our study, the cases with the introduction of systemic chemotherapy showed a significant prolongation of OS compared with the cases with clinical success, suggesting that the improvement of prognosis cannot be achieved by merely aiming for clinical success in biliary drainage for malignant biliary obstruction. Previous studies on prognostic factors after biliary drainage for non-HPB cancer have been limited. Several studies suggest that ECOG-PS is associated with OS [6,21]. Although a significant difference was not observed in this study, the introduction rate of systemic chemotherapy was low (*p* = 0.058) and OS (*p* = 0.089) was poor in cases with poor ECOG-PS. However, there were cases where ECOG-PS improves after biliary drainage, making it possible to introduce systemic chemotherapy. Therefore, a comprehensive assessment of the indications of biliary drainage is required.

Moreover, low serum CRP levels (<3.4 mg/dL) and biliary obstruction due to lymph node metastasis were the predictive factors for the introduction of systemic chemotherapy. CRP has also been considered a prognostic biomarker for malignant tumors [22]. Furthermore, several studies have reported that CRP is useful for predicting the efficacy and prognosis of systemic chemotherapy for cases with gastrointestinal cancer [23,24]. In our study, CRP levels did not affect the normalization rate of serum bilirubin levels, whereas serum albumin levels were lower in the high CRP group. These results suggest that the decrease in the introduction of systemic chemotherapy in cases with high CRP levels may be associated with a poor general condition rather than the efficacy of biliary drainage. In contrast, biliary drainage was highly effective regardless of serum albumin level in cases with biliary obstruction due to lymph node metastasis. There are a few reports focusing on the obstruction site. Kastelijn et al. reported that cases with biliary obstruction due to liver metastasis had poor biliary drainage [25]. Moreover, the presence of peritoneal dissemination worsens the prognosis and hinders the introduction of systemic chemotherapy in most types of cancer [26]. Therefore, aggressive biliary drainage with the aim of introducing systemic chemotherapy is desirable in cases with low CRP levels and biliary obstruction due to lymph node metastasis.

Because of the limited number of cases with biliary obstruction due to non-HPB cancer, there have been few comparisons between different drainage techniques. In a previous study of 55 cases with non-HPB cancer treated with ERCP, the drainage success rate was 91.7%, and biliary drainage was associated with a prolongation of OS [6], which is consistent with our study. There have been several reports on the efficacy of percutaneous transhepatic biliary drainage (PTBD) for biliary obstruction due to non-HPB cancer. PTBD for biliary obstruction associated with gastric cancer achieved favorable outcomes with a drainage success rate of 80–100% [27,28], whereas PTBD for colorectal cancer had a poor drainage success rate of only 50% [25]. Recently, endoscopic ultrasound-guided biliary drainage (EUS-BD) has become more frequently employed [29]. The usefulness of EUS-BD for biliary obstruction due to non-HPB cancer has also been reported [30]. Ogura et al. reported that EUS-BD was superior to PTBD in both clinical success and safety in biliary obstruction due to gastric cancer [30]. Endoscopic biliary drainage is often difficult in case with malignant hilar biliary obstruction or duodenal invasion [2,17,31,32]. In our study, neither of these factors was predictive of the clinical success and OS. However, EUS-BD is a promising option in cases where biliary drainage by ERCP is difficult under these conditions. EUS-BD is not a procedure that can be performed at all facilities due to its difficulty and complications. In fact, there were very few cases in which EUS-BD was performed for biliary obstruction due to non-HPB cancer during the observation period of this study, making it difficult to compare it with ERCP. No comparative study has been conducted between ERCP and EUS-BD for biliary obstruction due to non-HPB cancer. Therefore, a comparison of the efficacy and safety of ERCP and EUS-BD for biliary obstruction due to non-HPB cancer is required.

The superiority of SEMS in the prolongation of OS through the high rate of clinical success and the low incidence of stent occlusion has been reported for biliary obstruction in HPB cancer [15,28,29]. However, the placement of SEMS did not contribute to the clinical success or the prolongation of OS in our study. The median survival time was approximately half that of the previous reports in HPB cancer [17,33,34], and many cases died without RBO. Therefore, we believe that SEMS could not demonstrate the advantage of a long stent patency because of the short survival time in this study. Since it is expected that the advances in drug therapy improve the prognosis of non-HPB cancer, future investigation of the superiority of SEMS in a large number of cases is required.

This study has some limitations. First, this was a retrospective study with a limited number of cases, resulting in low statistical power. Although biliary obstruction due to non-HPB cancer is extremely rare compared to HPB cancer, further studies with a larger number of cases are required in the future. Second, considering the small number of cases, we investigated the cases without distinguishing the obstruction site or the stent type. We believe that these factors should be analyzed separately; however, these factors did not affect the clinical success or the prognosis in this study. Therefore, we believe that they have little impact on the validity of the results. Third, despite TRBO is important in evaluating the efficacy of biliary stent placement [11], TRBO in this study may be underestimated because many cases died without RBO. This study could not demonstrate the superiority of SEMS in biliary obstruction due to non-HPB cancer. In long-term survivors, SEMS may contribute to prolonged stent patency, but further investigation is required.

We believe that this study will provide useful insights for future research. It is not easy to collect a large number of cases of biliary obstruction due to non-HPB cancer because it is extremely rare compared to biliary obstruction due to HPB cancer. However, advances in systemic chemotherapy have improved the prognosis of various cancer types, and the number of patients with long-term survival is expected to increase in the future. Therefore, these results should be validated in a larger multicenter prospective study.

## 5. Conclusions

This study demonstrated that transpapillary drainage with ERCP is effective and safe for biliary obstruction due to non-HPB cancer. Cases with high serum albumin levels or the introduction of systemic chemotherapy showed a prolongation of OS, and the low CRP levels and the biliary obstruction due to lymph node metastasis were associated with the introduction of systemic chemotherapy. Serum albumin and CRP levels may be useful markers for predicting prognosis and determining treatment options in patients with biliary obstruction due to non-HPB cancer. We believe that aggressive biliary drainage should be considered to enable the introduction of systemic chemotherapy in cases with a promising prognosis.

## Figures and Tables

**Table 1:** Baseline patient characteristics.

Variable	Results
Age [years], median (total range)	66 (41–87)
Male gender, *n* (%)	26 (60%)
ECOG-PS, *n* (%)	
0 or 1	13 (30%)
≥2	30 (70%)
Primary disease, *n* (%)	
Colorectal cancer	18 (42%)
Gastric cancer	10 (23%)
Breast cancer	4 (9%)
Uterine cancer	3 (7%)
Malignant lymphoma	3 (7%)
Lung cancer	2 (5%)
Ovarian cancer	2 (5%)
Salivary gland cancer	1 (2%)
Cause of obstruction, *n* (%)	
Peritoneal dissemination	14 (33%)
Liver metastasis	13 (30%)
Lymph node metastasis	12 (28%)
Pancreatic metastasis	4 (9%)
Duodenal invasion, *n* (%)	16 (37%)
Obstruction site, *n* (%)	
Distal bile duct	27 (63%)
Hilar bile duct	16 (37%)
Undergoing chemotherapy, *n* (%)	22 (51%)
Laboratory data at biliary drainage, median (total range)	
Total bilirubin [mg/dL]	7.0 (1.0–33.4)
AST [U/L]	126 (42–653)
ALT [U/L]	138 (23–678)
ALP [U/L]	736 (254–2998)
Albumin [g/dL]	3.1 (1.3–4.4)
WBC [/μL]	7180 (2830–24550)
CRP [mg/dL]	3.4 (0.3–31.3)
Total number of biliary drainage procedures, *n* (%)	
Once	26 (60%)
Twice	11 (26%)
Three times	6 (14%)
Implementation of EST, *n* (%)	26 (60%)
Stent type, *n* (%)	
PS	27 (63%)
SEMS	16 (37%)

Abbreviations: ALP, alkaline phosphatase; ALT, alanine aminotransferase; AST, aspartate aminotransferase; CRP, C-reactive protein; ECOG-PS, Eastern Cooperative Oncology Group Performance Status; EST, endoscopic sphincterotomy; PS, plastic stents; SEMS, self-expandable metal stents; WBC, white blood cell.

**Table 2:** Clinical outcomes.

	Results
Technical success, *n* (%)	43 (100%)
Clinical success, *n* (%)	35 (81%)
RBO, *n* (%)	14 (33%)
Cause of RBO	
Tumor ingrowth, *n* (%)	5 (12%)
Tumor over growth, *n* (%)	5 (12%)
Sludge, *n* (%)	4 (9%)
Non-RBO adverse events	
Pancreatitis, *n* (%)	5 (12%)
Overall survival from biliary drainage [days], median (total range)	63 (8–1140)
Death without RBO, *n* (%)	26 (60%)
TRBO [days], median (total range)	176 (11–245)

Abbreviations: RBO, recurrent biliary obstruction; TRBO, time to recurrent biliary obstruction.

**Table 3:** Patient characteristics in the cases with clinical success and the cases without clinical success.

Variable	Clinical Success Group (*n* = 35)	Non-Clinical Success Group (*n* = 8)	*p*-Value
Age [years], median (total range)	66 (41–87)	66 (47–81)	0.925
Male gender, *n* (%)	20 (57%)	6 (75%)	0.446
ECOG-PS, *n* (%)			
0 or 1	10 (29%)	3 (38%)	0.681
≥2	25 (71%)	5 (62%)	
Primary disease, *n* (%)			
Colorectal cancer	15 (43%)	3 (38%)	1.000
Gastric cancer	7 (20%)	3 (38%)	0.362
Breast cancer	3 (9%)	1 (12%)	1.000
Uterine cancer	3 (9%)	0 (0%)	1.000
Malignant lymphoma	3 (9%)	0 (0%)	1.000
Lung cancer	1 (2%)	1 (12%)	0.341
Ovarian cancer	2 (6%)	0 (0%)	1.000
Salivary gland cancer	1 (2%)	0 (0%)	1.000
Cause of obstruction, *n* (%)			
Peritoneal dissemination	10 (29%)	4 (50%)	0.404
Liver metastasis	10 (29%)	3 (38%)	0.681
Lymph node metastasis	12 (33%)	0 (0%)	0.082
Pancreatic metastasis	3 (9%)	1 (12%)	1.000
Duodenal invasion, *n* (%)	14 (40%)	2 (25%)	0.688
Obstruction site, *n* (%)			
Distal bile duct	24 (69%)	3 (38%)	0.125
Hilar bile duct	11 (31%)	5 (62%)	
Laboratory data, median (total range)			
Total bilirubin [mg/dL]	6.1 (1.0–33.4)	11.2 (7.0–15.2)	0.083
AST [U/L]	133 (42–587)	106 (64–653)	0.731
ALT [U/L]	153 (23–647)	110 (64–678)	0.532
ALP [U/L]	744 (255–2998)	714 (397–1172)	0.890
Albumin [g/dL]	3.1 (1.3–4.4)	2.6 (2.1–3.5)	0.146
WBC [/μL]	7180 (3230–21940)	7420 (2830–24550)	1.000
CRP [mg/dL]	3.5 (0.3–31.3)	3.2 (1.0–19.9)	0.710
Stent type, *n* (%)			
PS	21 (60%)	6 (75%)	0.688
SEMS	14 (40%)	2 (25%)	

Abbreviations: ALP, alkaline phosphatase; ALT, alanine aminotransferase; AST, aspartate aminotransferase; CRP, C-reactive protein; ECOG-PS, Eastern Cooperative Oncology Group Performance Status; PS, plastic stents; SEMS, self-expandable metal stents; WBC, white blood cell.

**Table 4:** Univariate and multivariate analyses for the introduction of systemic chemotherapy in patient characteristics.

Variable	Categories	Univariate Analysis			Multivariate Analysis		
		OR	95% CI	*p*-Value	OR	95% CI	*p*-Value
Age [years]	≥65 vs. <65	1.22	0.36–4.21	0.748			
Sex	Male vs. female	0.89	0.26–3.11	0.859			
ECOG-PS	≥2 vs. 0 or 1	0.27	0.07–1.05	0.058			
Primary disease	Colorectal cancer vs. other cancer	0.64	0.18–2.24	0.482			
	Gastric cancer vs. other cancer	0.58	0.13–2.66	0.484			
Cause of obstruction	Lymph node metastasis vs. others	4.89	1.17–20.40	0.030	8.71	1.34–56.50	0.023
Duodenal invasion	Positive vs. negative	0.83	0.25–3.11	0.457			
Obstruction site	Distal bile duct vs. hilar bile duct	4.67	1.08–20.20	0.039	4.60	0.86–24.70	0.075
Total bilirubin [mg/dL]	>7.0 vs. ≤7.0	0.34	0.10–1.21	0.097			
AST [U/L]	<126 vs. ≥126	0.60	0.17–2.06	0.418			
ALT [U/L]	<138 vs. ≥138	0.60	0.17–2.06	0.418			
ALP [U/L]	<736 vs. ≥736	1.95	0.56–6.73	0.292			
Albumin [g/dL]	>3.1 vs. ≤3.1	3.21	0.90–11.50	0.073			
WBC [/μL]	>7180 vs. ≤7180	0.40	0.11–1.41	0.155			
CRP [mg/dL]	<3.4 vs. ≥3.4	4.27	1.13–16.10	0.032	8.62	1.50–49.30	0.016
Stent type	SEMS vs. PS	2.00	0.56–7.09	0.283			

The cut-off values for laboratory data were the median value of all cases. Abbreviations: CI, confidence interval; CRP, C-reactive protein; ECOG-PS, Eastern Cooperative Oncology Group Performance Status; HR, hazard ratio; PS, plastic stents; SEMS, self-expandable metal stents; WBC, white blood cell.

**Table 5:** Univariate and multivariate analyses of prognostic factors for overall survival.

Variable	Categories	Univariate Analysis			Multivariate Analysis		
		HR	95% CI	*p*-Value	HR	95% CI	*p*-Value
Age [years]	≥65 vs. <65	1.15	0.61–2.17	0.667			
Sex	Male vs. female	1.62	0.85–3.10	0.146			
ECOG-PS	≥2 vs. 0 or 1	1.85	0.91–3.76	0.089			
Primary disease	Colorectal cancer vs. other cancer	1.81	0.94–3.49	0.078			
	Gastric cancer vs. other cancer	1.12	0.54–2.32	0.755			
Cause of obstruction	Lymph node metastasis vs. others	0.55	0.26–1.17	0.122			
Duodenal invasion	Positive vs. negative	1.28	0.67–2.45	0.457			
Obstruction site	Distal bile duct vs. hilar bile duct	0.74	0.38–1.41	0.359			
Total bilirubin [mg/dL]	>7.0 vs. ≤7.0	1.49	0.79–2.81	0.214			
AST [U/L]	<126 vs. ≥126	0.97	0.52–1.83	0.932			
ALT [U/L]	<138 vs. ≥138	0.98	0.52–1.85	0.949			
ALP [U/L]	<736 vs. ≥736	0.52	0.27–0.99	0.046	0.66	0.32–1.34	0.248
Albumin [g/dL]	>3.1 vs. ≤3.1	0.40	0.21–0.78	0.007	0.44	0.22–0.91	0.026
WBC [/μL]	>7180 vs. ≤7180	1.22	0.65–2.30	0.545			
CRP [mg/dL]	<3.4 vs. ≥3.4	0.46	0.24–0.88	0.018	0.69	0.35–1.36	0.281
Treatment after drainage	Systemic chemotherapy vs. Best supportive care	0.24	0.12–0.48	<0.001	0.23	0.11–0.49	<0.001
Stent type	SEMS vs. PS	0.73	0.38–1.41	0.350			

The cut-off values for laboratory data were the median value of all cases. Abbreviations: CI, confidence interval; CRP, C-reactive protein; ECOG-PS, Eastern Cooperative Oncology Group Performance Status; HR, hazard ratio; PS, plastic stents; SEMS, self-expandable metal stents; WBC, white blood cell.

## Data Availability

The data presented in this study are available on request from the corresponding author. The data are not publicly available due to privacy.

## Abbreviations

The following abbreviations are used in this manuscript:

ERCP	Endoscopic retrograde cholangiopancreatography
HPB	Hepato-pancreato-biliary
PS	Plastic stents
SEMS	Self-expandable metal stents
UCSEMS	Uncovered SEMS
FCSEMS	Fully covered SEMS
EST	Endoscopic sphincterotomy
RBO	Recurrent biliary obstruction
TRBO	Time to RBO
AEs	Adverse events
OS	Overall survival
CRP	C-reactive protein
ECOG-PS	Eastern Cooperative Oncology Group Performance Status
AST	Aspartate aminotransferase
ALT	Alanine aminotransferase
